# Effect of Electrochemical Synthesis Parameters on the Morphology, Crystal and Chemical Structure, and Sorption Efficiency of Basic Bismuth Nitrates

**DOI:** 10.3390/molecules30051020

**Published:** 2025-02-23

**Authors:** Slobodan M. Najdanović, Miloš M. Kostić, Milica M. Petrović, Nena D. Velinov, Miljana D. Radović Vučić, Jelena Z. Mitrović, Aleksandar Lj. Bojić

**Affiliations:** Department of Chemistry, Faculty of Sciences and Mathematics, University of Niš, Višegradska 33, 18000 Niš, Serbia; milica.petrovic3@pmf.edu.rs (M.M.P.); nena.velinov@pmf.edu.rs (N.D.V.); miljana.radovic@pmf.edu.rs (M.D.R.V.); jelena.mitrovic1@pmf.edu.rs (J.Z.M.); aleksandar.bojic@pmf.edu.rs (A.L.B.)

**Keywords:** electrodeposition, basic bismuth nitrates, textile dyes, Reactive Blue 19, sorption, water treatment

## Abstract

Bismuth basic nitrates (BBNs) were synthesized via an electrochemical method, i.e., by electrodeposition from an acidic solution of bismuth nitrate, followed by thermal treatment in an air environment. For the first time, the influence of various electrochemical parameters on the morphology, crystal structure, and chemical structure of BBNs was examined. The following synthesis parameters were investigated: electrodeposition current density, thermal treatment temperature of the obtained deposit, and working electrode material (cathode). The obtained materials were characterized by SEM-EDX, XRD, FTIR, TG, and N_2_ adsorption/desorption methods and were applied for the sorption of the textile dye RB19. The results showed that the electrodeposition current density and thermal treatment temperature affect the surface morphology, chemical composition, and crystal structure of the obtained materials, as well as the RB19 sorption efficiency. On the other hand, the working electrode material does not affect the properties of the synthesized materials mentioned. Kinetic, isotherm, and thermodynamic analysis of the sorption process were also examined.

## 1. Introduction

Approximately fifteen different BBNs were described in the literature up to the 17th Century. However, the structures of only a few of these compounds have been unequivocally confirmed. The main reasons for this are the great variety of compounds, the difficulty in isolating pure phases, the presence of very weakly bound water molecules or hydroxyl groups, and the lack of sufficiently powerful methods for identification. Therefore, only a few BBNs have been fully characterized [1,2].

BBNs are most often obtained by hydrolysis of bismuth nitrates. By analyzing the crystal structure of these materials, it was determined that they mainly consist of complex ions, i.e., polycations of the general formula [Bi_6_O_x_(OH)_8−x_]^(10−x)+^, among which the most common are polycations [Bi_6_O_4_(OH)_4_]^6+^ and [Bi_6_O_5_(OH)_3_]^5+^. The polycation of the above general formula is formed by intramolecular polycondensation of the cation [Bi_6_(OH)_12_]^6+^ [1,2,3].

BBNs are used in medicine. Their first use as a medicine dates back to the Middle Ages, and were first published in the literature in 1786, where they were listed under the names *magisterium bismuti* and *bismutum subnitricum* and were used to treat dyspepsia [4]. The absorption of the so-called bismuth subnitrate in the skin, stomach, and intestines is very weak due to its very poor solubility, but it reacts with hydrochloric acid in the stomach. In addition, bismuth subnitrate covers the gastric mucosa and thus protects it. In the 19th Century, bismuth subnitrate was found to be useful for wound healing, and in the 20th Century, it was also an antisyphilitic agent. Today, bismuth subnitrate is used as a pharmaceutical ingredient in drugs to relieve stomach pain caused by the bacteria *Helicobacter pylori* [4]. In medicine, BBNs are also used as antiseptics [3]. Additionally, BBNs are used to prepare Dragendorff’s reagent, which is used to detect alkaloids, giving yellow, orange, red, or brown color reactions, depending on the nature of the alkaloid [5]. Bismuth subnitrate iodoform paraffin paste is used in the management of inflammatory follicular cyst [6], as a precursor for the synthesis of medications [7], and in veterinary medicine [8].

Due to their low solubility, BBNs are used to remove halides and oxo anions by ion exchange reactions [9]. In addition, they are used as precursors for the production of bismuth oxide [2,10]. BBNs have been found to be an excellent catalyst for organic synthesis reactions [11,12]. They have also been used as photocatalysts in many studies [13,14,15,16,17,18,19,20]. The use of BBNs as sorbents is very rare and has been investigated in only a few scientific studies [9,21,22,23,24].

The innovation in this study is that, for the first time, the influence of electrochemical synthesis parameters for obtaining BBNs has been examined in detail. The aim of this study was to examine the effects of electrochemical synthesis parameters on morphology, crystal structure, and chemical structure, as well as the sorption performance of BBNs. The obtained materials were fully characterized by scanning electron microscopy (SEM), X-ray diffraction (XRD), energy dispersive X-ray spectroscopy (EDX), Fourier transform infrared spectroscopy (FTIR), Thermogravimetric analysis (TG) and N_2_ adsorption/desorption methods. Materials were used to remove the textile dye Reactive Blue 19 (RB19) from water. To obtain the sorbent with the best properties (highest sorption capacity and rate of achieving equilibrium), the following parameters of electrochemical synthesis were optimized: electrodeposition current density, working electrode material, and thermal treatment temperature of the obtained deposit. There are no studies in the literature on the influence of the electrochemical parameters of synthesis on the properties of the obtained BBNs. The study of the influence of the electrochemical synthesis parameters of these materials and their influence on their sorption performance is novel in this field of science. This could be of scientific interest, and we hope that this research will further expand in this area and bring new knowledge in the near future.

## 2. Results and Discussion

### 2.1. Characterization of the Materials

#### 2.1.1. SEM Analysis

**Figure 1:**
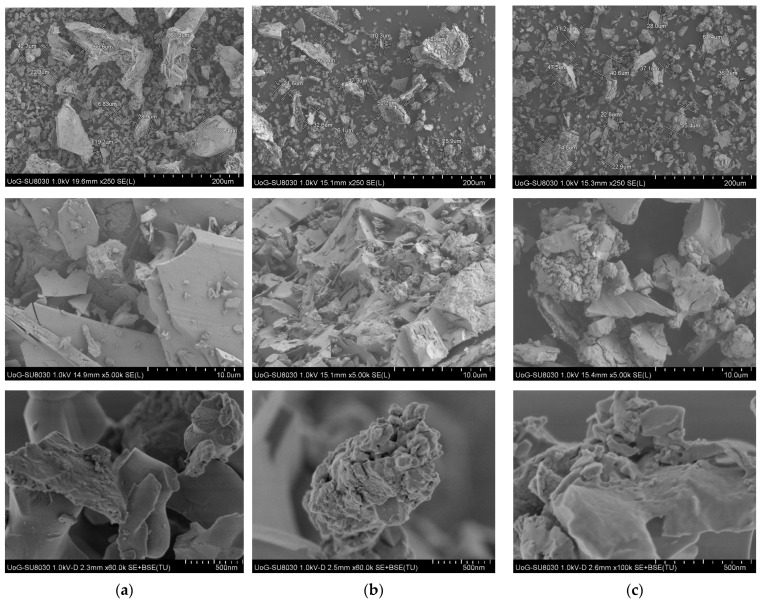
SEM micrographs of materials obtained at different current densities: (**a**) 50 mA cm^−2^, (**b**) 100 mA cm^−2^, and (**c**) 200 mA cm^−2^.

The morphological structure of the synthesized materials at different current densities is presented in Figure 1. The results showed that the material synthesized at a low current density (50 mA cm^−2^) had the highest crystallinity. This material consists of small particles aggregated into larger crystals. The crystals have different irregular shapes, and their surfaces are smooth. Small particles can be observed on the surface of the crystal.

With an increase in the current density during the process of electrochemical synthesis, materials with lower crystallinity are obtained. At a current density of 100 mA cm^−2^, large crystals are not fully formed. The process of formation of large crystals is not complete, most likely because at a higher current density, the deposition rate and amount of deposited material on the cathode are greater, so it takes more time to form crystals. At a current density of 200 mA cm^−2^, large crystals are rare. The material is composed of small crystals that are sintered with each other. The reason for such a structure is the fast growth of crystals; in this case, it is impossible to form regular shapes.

The morphology of the material does not change significantly after thermal treatment at 350 °C or 500 °C. Additionally, the cathode material did not affect the morphological structure. Therefore, these results are not reported in the manuscript.

#### 2.1.2. XRD Analysis

**Figure 2:**
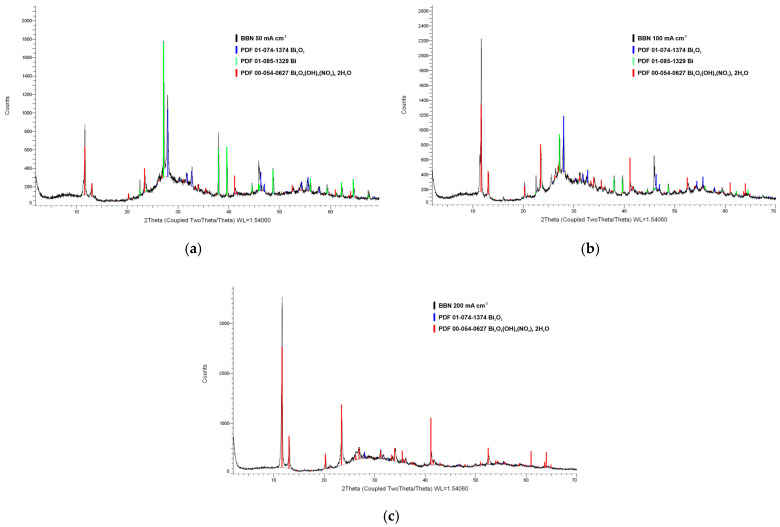
XRD patterns of materials synthesized at current densities of (**a**) 50 mA cm^−2^, (**b**) 100 mA cm^−2^, and (**c**) 200 mA cm^−2^.

Figure 2 shows the XRD patterns of the materials synthesized at different current densities. Materials are composed of several compounds. A list of the compounds and their weight percentages is presented in Table 1.

The materials synthesized at different current densities, from 50 to 200 mA cm^−2^, are mainly composed of rhombohedral with hexagonal axes Bi_6_O_5_(OH)_3_(NO_3_)_5_⋅2H_2_O (space group R (0), a = 15.18500, c = 15.83400, a/b = 1.00000, c/b = 1.04274). The materials are also composed of tetragonal Bi_2_O_3_ (space group C-42b (117), a = 10.93000, c = 5.62000, a/b = 1.00000, c/b = 0.51418). Metallic bismuth, rhombohedral with hexagonal axes (space group R-3m (166), a = 4.54600, c = 11.86200, a/b = 1.00000, c/b = 2.60933), is present in materials obtained at lower current densities of 50 and 100 mA cm^−2^.

The results of the quantitative analysis showed that the weight percent of BBN increased from 41.48% for the material obtained at 50 mA cm^−2^ to 98.34% for the material obtained at 200 mA cm^−2^. Additionally, the weight percentages of bismuth and bismuth oxide decreased. Different composition of synthesized material at different current densities is due to the fact that the oxidation of the material is not full at low current densities. Oxidation on the cathode occurs when the concentration of H^+^ ions decreases significantly because diffusion is slow, followed by the reduction of H_2_O and the production of OH^−^. Then, the local pH around the cathode increases, so the probability that the incoming unreduced Bi(III) ions “collide” with OH^−^ ions increases and, in the presence of NO_3_^−^ ions, form basic nitrates instead of being reduced at the cathode to metallic bismuth.

**Figure 3:**
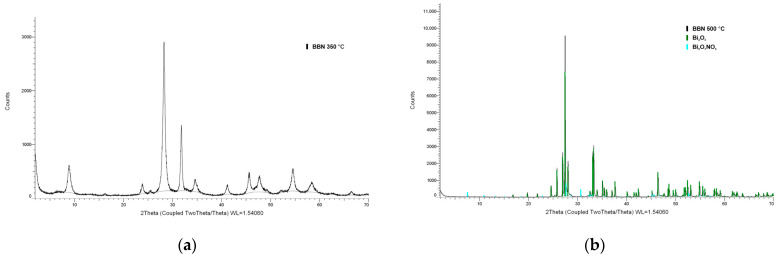
XRD patterns of materials synthesized at (**a**) 350 °C and (**b**) 500 °C.

Figure 3 shows the XRD patterns of the materials obtained at different temperatures during thermal treatment. The XRD pattern of the material obtained at 350 °C does not match any XRD pattern from the available databases. Based on the obtained peaks, it can be concluded that this compound is some form of BBN. The material synthesized at 500 °C is mainly composed of monoclinic bismuth oxide Bi_2_O_3_ (space group P21/c (14), a = 5.84440, b = 8.15740, c = 7.50320, a/b = 0.71645, and c/b = 0.91980). The minor phase was orthorhombic bismuth oxide nitrate (space group Cmca (64), a = 16.28000, b = 5.54800, c = 23.30100, a/b = 2.93439, c/b = 4.19989). The quantitative analysis showed that the weight percent of Bi_2_O_3_ was 92.26%, and that of Bi_5_O_7_NO_3_ was 7.74%. Based on the obtained results, it can be concluded that by thermal treatment of the obtained deposit at 500 °C, the largest percentage of Bi_6_O_5_(OH)_3_(NO_3_)_5_⋅2H_2_O oxidizes to Bi_2_O_3_, and the rest of the BBN changes its form to Bi_5_O_7_NO_3_.

The materials obtained on a stainless-steel cathode have the same crystal structure as the materials obtained on a titanium cathode. Therefore, these results are not presented in the manuscript.

#### 2.1.3. EDX Analysis

The results of the EDX analysis are presented in Table 2. All the materials consisted of the following elements: Bi, O, and N. The results of the EDX analysis were consistent with the results obtained by the XRD analysis. With increasing electrodeposition current density, the weight percent of Bi decreases, while the weight percent of O and N increases. This occurs because metallic bismuth oxidizes in a higher percentage to Bi_6_O_5_(OH)_3_(NO_3_)_5_⋅2H_2_O at higher current densities. With an increase in the temperature of the thermal treatment to 500 °C, the mass fraction of Bi increases as the material almost completely oxidizes to Bi_2_O_3_. The material obtained at 350 °C probably consisted of Bi_2_O_3_ and some form of BBN.

#### 2.1.4. FTIR Analysis

**Figure 4:**
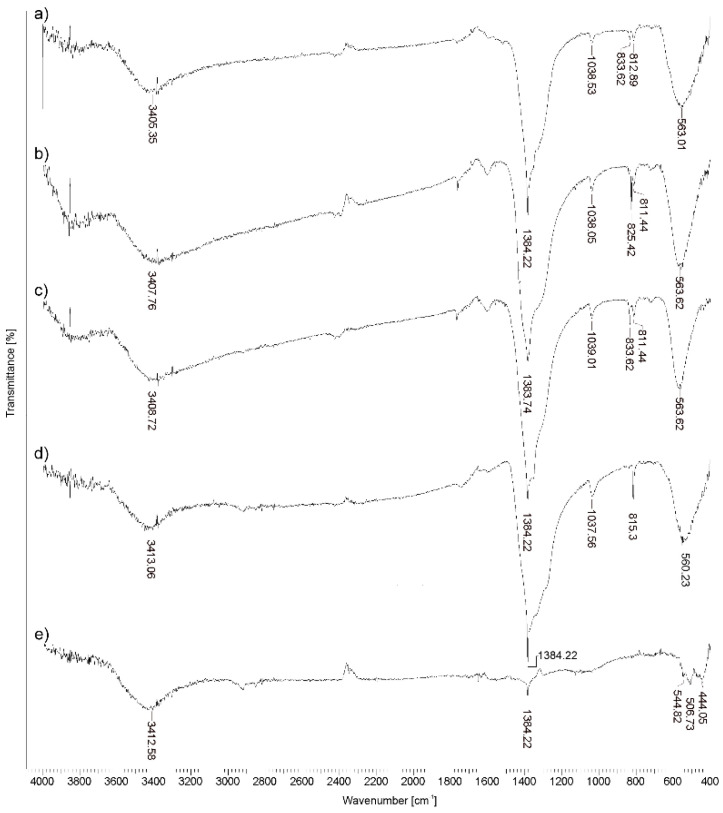
FTIR spectra of materials synthesized at current densities of (**a**) 50 mA cm^−2^, (**b**) 100 mA cm^−2^, (**c**) 200 mA cm^−2^, (**d**) 350 °C, and (**e**) 500 °C.

FTIR spectra of materials synthesized at current densities of 50 mA cm^−2^, 100 mA cm^−2^, and 200 mA cm^−2^ are shown in Figure 4a–c, respectively. For all three materials, the peaks are located at the characteristic wavenumbers for BBN Bi_6_O_5_(OH)_3_(NO_3_)_5_⋅2H_2_O described by Christensen et al. [3]. The FTIR spectra of all materials have a strong broadband at around 3430 cm^−1^, corresponding to the stretching vibration of the –OH group. The bands at around 1384 cm^−1^, 1038 cm^−1^, and 811 cm^−1^ are attributed to the vibrations of NO_3_^−^ [25,26,27]. The band at around 563 cm^−1^ can be assigned to the stretching vibrations of the Bi–O bond [23]. On the FTIR spectra, the bands characteristic of BBN predominate as the obtained materials are mainly composed of this compound.

By comparing the FTIR spectra of materials obtained at temperatures of 200 °C, 350 °C, and 500 °C at Figure 4c–e, it can be seen that at lower temperatures BBN is formed, while at a temperature of 500 °C, the bands characteristic of BBN almost completely disappeared. The FTIR spectrum of the material synthesized at 500 °C shows a large decrease in the intensity of the peak at 1384 cm^−1^, which means that the proportion of BBN in the materials has been significantly reduced. Also, the bands in the region between 444 and 544 cm^−1^, which can be attributed to Bi–O stretching vibrations, in the FTIR spectrum of this material confirm the synthesis of Bi_2_O_3_ [28,29]. The results of FTIR analysis are consistent with the results obtained by XRD analysis.

#### 2.1.5. TG Analysis

The TG analysis of materials synthesized at 200 °C, 350 °C, and 500 °C was performed and the results are presented in Figures S1–S3, respectively. The results of the TG analysis confirmed the results obtained by the XRD analysis and FTIR analysis. With increasing thermal treatment temperature, weight loss decreases. This confirms that at the lowest temperature, Bi_6_O_5_(OH)_3_(NO_3_)_5_⋅2H_2_O was obtained in the highest percentage, while with the increase in temperature, BBNs with a higher percentage of Bi_2_O_3_ are obtained. By heating to a temperature of 600 °C, the mass of the material obtained at 500 °C decreases by only 0.8%. This means that the material obtained at 500 °C has the highest percentage of pure Bi_2_O_3_ and therefore has almost no weight loss.

#### 2.1.6. N_2_ Adsorption/Desorption Method

The textural properties of the materials, such as the specific surface area, pore volume, area, and diameter, were determined via the N_2_ adsorption/desorption method. The obtained results are shown in Table 3.

The results show that all materials have very small specific surface areas and pore volumes, so no important conclusions can be drawn by comparing the obtained results. Therefore, it can be concluded that the electrodeposition current density and the thermal treatment temperature do not affect the textural properties of the material. Also, the working electrode material (titanium or stainless-steel) on which the electrodeposition was performed did not affect the textural characteristics of the material. Since similar results were obtained in both cases, when using both titanium and stainless-steel cathodes, only the results for materials obtained on titanium electrodes are shown in Table 3.

### 2.2. Sorption Efficiency of the Obtained Materials for the Removal of RB19

#### 2.2.1. Effect of pH on the Removal Efficiency of RB19

**Figure 5:**
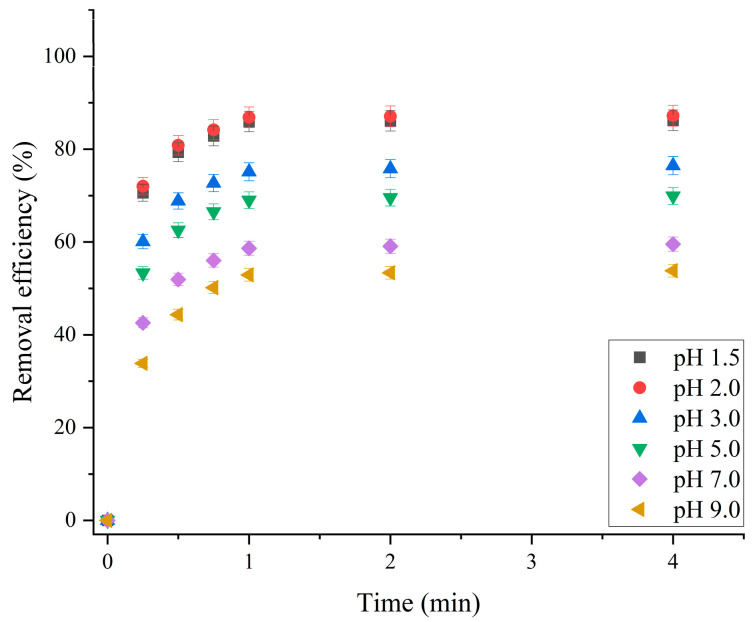
The effect of pH on the removal efficiency of RB19: sorbent dose, 500.0 mg dm^−3^; initial RB19 concentration, 600.0 mg dm^−3^; stirring speed, 200 rpm; and temperature, 25.0 ± 0.5 °C.

The surface charge of a material has a large influence on its sorption performance. The pH of the solution affects the surface charge of the sorbent and the degree of ionization of the sorbate, thus affecting the efficiency of the sorption process [30]. Therefore, in order to make a comparison of the sorption efficiencies of the obtained materials relevant, the effect of pH was studied, and the optimal pH value was determined, at which further experiments were performed. The results showed that the effect of pH on the removal of RB19 was the same for all the obtained materials. Therefore, only the results for the material obtained at 200 mA cm^−2^ at 200 °C are presented (Figure 5). The effect of pH on the removal efficiency of RB19 was investigated in the pH range from 1.5 to 9.0. An extremely high concentration of the dye RB19 (600.0 mg dm^−3^) was used in the experiments to make it easier to determine the difference in removal efficiency at different pH values.

The results showed that the highest removal efficiency was achieved at solution pH values of 1.5 and 2.0 and that the removal efficiency decreased by increasing pH up to 9.0. By changing the pH of the solution from 2.0 to 9.0, the removal efficiency decreased from 87.27% to 53.79%. At a solution pH of 1.5, the material removal efficiency is slightly lower than that at a pH of 2.0.

Since sulfonic acids are strong acids, the sulfonic groups of RB19 will be in dissociated form, i.e., deprotonated (–SO^3−^), over the entire pH range. In a strongly acidic environment (pH 1.5–2.0), the surface of the sorbent is positively charged because the pI of the BBN sorbent is 2.12. Then, the electrostatic attraction occurs between the sulfonic groups of the RB19 dye, which are anionic, and the surface of the sorbent is the strongest [31]. Additionally, at low pH values, the oxygen atoms (Bi–O and Bi–OH) in polycation [Bi_6_O_5_(OH)_3_]^5+^ are protonated, so the electron density around adjacent bismuth atoms decreases. In this way, the chemical interaction (coordination bond, most likely bidentate bond of sulfonic groups with bismuth atoms) between the bismuth atoms of the sorbent and the sulfonic group of the RB19 dye is improved. Therefore, the efficiency of the BBN sorbent for removing RB19 dye is highest at pH ≤ 2.0 and decreases with increasing pH toward basic values because the electrostatic attraction and the strength of the coordination bond between Bi atoms and –SO_3_^−^ ions weaken [23].

Therefore, the material has the highest sorption capacity for RB19 at pH ≤ 2.0, and this capacity decreases with increasing pH (Figure 5). The maximum sorption capacity at pH 2.0 is 1047.24 mg g^−1^. Furthermore, the data in Figure 5 show that over the whole pH range investigated, the sorption capacity is very high, being 645.48 mg g^−1^ at pH 9.0, where the efficiency is the lowest. Thus, the high sorption capacity of the obtained materials over the whole investigated pH range can be considered a beneficial characteristic for the application of sorbent in water treatment, enabling it to be used without the necessity for pH adjustment and the addition of any other chemical component to the sorption system. Since the optimum pH is 2.0, all further experiments were performed at pH 2.0.

#### 2.2.2. Effect of the Cathode Material

To investigate the effect of the cathode material on the chemical and sorption properties of the material, titanium and stainless-steel cathodes of identical shapes and dimensions (10 × 20 mm plates) were used for electrodeposition. Other synthesis parameters included a bismuth nitrate concentration of 0.1 mol dm^−3^ (aqueous solution), a current density of 200 mA cm^−2^, an electrodeposition time of 3.5 min, a heat treatment temperature of 200 °C and a heat treatment time of 90 min.

**Figure 6:**
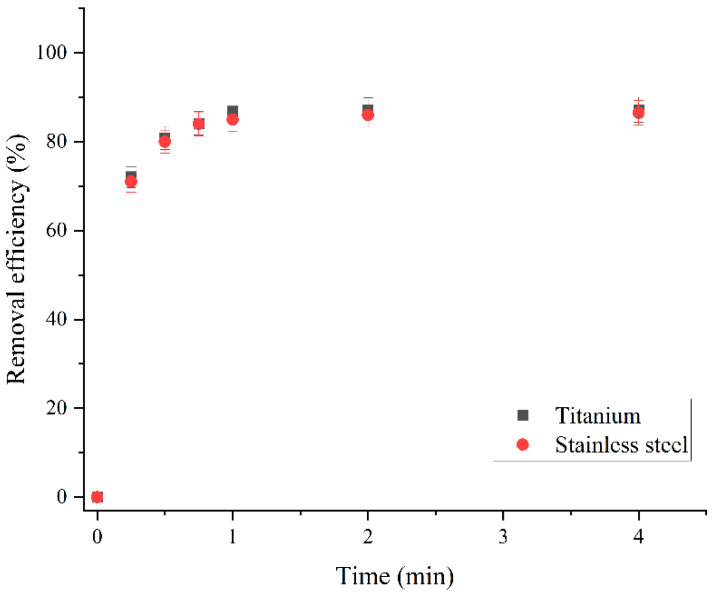
Comparison of RB19 dye removal efficiency using sorbents synthesized at titanium and stainless-steel cathodes: initial RB19 concentration, 600.0 mg dm^−3^; sorbent dose, 500.0 mg dm^−3^; pH, 2.0; stirring speed, 200 rpm; temperature, 25.0 ± 0.5 °C.

By comparing the removal efficiency of RB19, it can be concluded that the cathode material does not affect the sorption activity, as shown in Figure 6. After 60 s of sorption treatment, the removal efficiency was 87.27% for the sorbent synthesized on the titanium cathode and 86.50% for the stainless-steel cathode. Analysis of the chemical, crystalline, and morphological structure of these two materials showed that the change in the cathode material did not affect these properties either. However, titanium cathodes have several advantages over stainless-steel cathodes. Titanium has greater mechanical stability and electrochemical inertness. In contrast, the stainless-steel cathode partially dissolves at high current densities, so the material can become contaminated with iron atoms. Therefore, in this study, only materials obtained on the titanium cathode were further investigated.

#### 2.2.3. Effect of Electrodeposition Current Density

**Figure 7:**
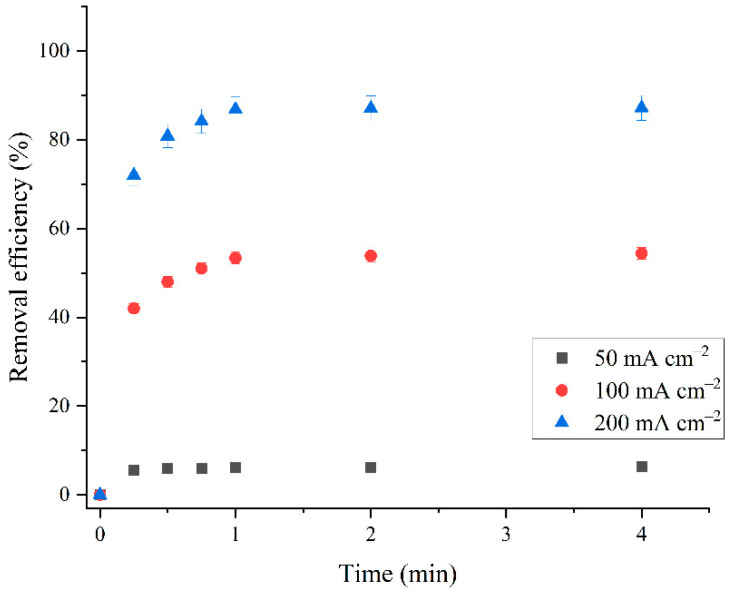
Comparison of RB19 dye removal efficiency using sorbents synthesized at 50, 100, and 200 mA cm^−2^: initial RB19 concentration, 600.0 mg dm^−3^; sorbent dose, 500.0 mg dm^−3^; pH, 2.0; stirring speed, 200 rpm; temperature, 25.0 ± 0.5 °C.

One of the main parameters of the electrochemical process is the electrodeposition current density. The optimal current density was determined by comparing the results of dye sorption on the obtained materials at a current density of 50 mA cm^−2^, 100 mA cm^−2^, and 200 mA cm^−2^. The electrodeposition times were 15.0, 7.5, and 3.75 min, respectively. With increasing current density, the electrodeposition time was proportionally reduced to obtain, according to Faraday’s law, the same amount of deposit in all cases. The sorption activity of the material increases with increasing current density during electrodeposition from 50 mA cm^−2^ to 200 mA cm^−2^ (Figure 7). The removal efficiency of RB19 with material obtained at a low electrodeposition current density (50 mA cm^−2^) was only 6.40%. As the current density of electrodeposition increases, the sorption performance of the material significantly increases. Material obtained at 100 mA cm^−2^ removed 54.40% of the dye, and material obtained at 200 mA cm^−2^ removed 87.27%. This can be explained by the much slower and more controlled deposition at the cathode at lower current densities; in doing so, the resulting coating consists of tiny crystals. In contrast, at high current densities, a coarse-grained deposit separates at the cathode, which contains, in addition to the crystalline phase, an amorphous phase. These are, most likely, aggregates formed by the fusion of many small crystals due to the excessive rate of deposition at the cathode. In addition, at high current densities, hydrogen is formed at the cathode, so the deposit acquires a spongy structure that allows complete oxidation of the separated deposit into BBN during thermal treatment. Additionally, at lower current densities, mostly metallic Bi is obtained, while at higher currents, BBNs are also obtained. Since materials obtained at lower current densities are not completely oxidized and metal Bi does not possess sorption activity, materials obtained at higher current densities have shown significantly better sorption abilities. A current density of 200 mA cm^−2^ was taken as the optimal current density since a further increase in the current density did not lead to an improvement in the sorption activity of the material.

#### 2.2.4. Effect of Temperature During the Thermal Treatment of the Obtained Deposit

The optimum material synthesis temperature was determined by thermal treating of the obtained deposit for 90 min at three different temperatures: 200 °C, 350 °C, and 500 °C. Thermal treatment was preceded by electrodeposition on titanium cathodes from an aqueous solution of bismuth nitrate at a concentration of 0.1 mol dm^−3^ at a constant current density of 200 mA cm^−2^ for 3.5 min.

**Figure 8:**
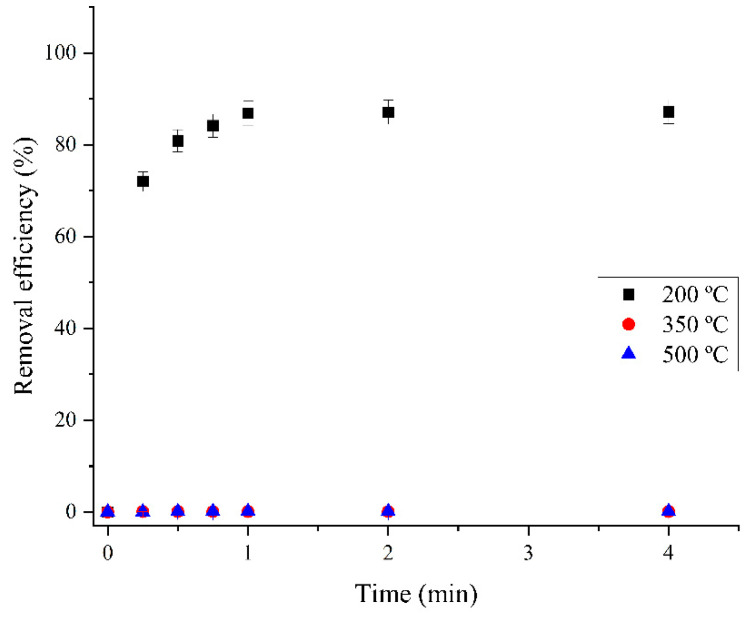
Comparison of RB19 dye removal efficiency using sorbents synthesized at 200, 350, and 500 °C: initial RB19 concentration, 600.0 mg dm^−3^; sorbent dose, 500.0 mg dm^−3^; pH, 2.0; stirring speed, 200 rpm; temperature, 25.0 ± 0.5 °C.

The efficiency of these materials for the removal of RB19 as a function of time is shown in Figure 8. The results showed that the material synthesized at 200 °C had a much higher sorption activity than the materials synthesized at 350 °C and 500 °C. The removal efficiency of RB19 with material synthesized at 200 °C was 87.27%, and with materials synthesized at 350 °C and 500 °C was slightly higher than zero because the materials obtained at 350 °C and 500 °C were oxidized to a higher percentage towards bismuth oxide, which has low sorption activity. Therefore, the optimal synthesis temperature is 200 °C.

### 2.3. Kinetic, Equilibrium and Thermodynamic Study

Kinetics, isotherms, and thermodynamics analysis were investigated only for the most efficient material, synthesized at 200 mA cm^−2^, 200 °C, and at titanium cathode.

#### 2.3.1. Kinetic Study

Sorption in the liquid–solid system has a heterogeneous character and takes place through several reaction and diffusion stages, of which the slowest stage determines the overall speed of the sorption process. By investigating the kinetics, the sorption mechanism can be determined, and the phase on which the sorption rate depends can be identified. To examine the mechanism controlling the rate of the sorption process, pseudo-first and pseudo-second order kinetic models were used. The results of fitting experimental data by these models are shown in Figure S4. Kinetic parameters obtained by nonlinear regression analysis are presented in Table 4.

The results obtained by both pseudo-first and pseudo-second order models have good agreement with experimental results. The determination coefficient for both models is very high (higher than 0.99). However, if the *MRD* is compared, the pseudo-second order shows better agreement with the experimental results. *MRD* of the pseudo-first order is 1.85, while *MRD* of the pseudo-second order is 0.69, meaning that the sorption of RB19 dye follows a pseudo-second order model, i.e., implies that the sorption process is controlled by the interaction between the sorbate and specific binding sites on the sorbent.

#### 2.3.2. Isotherm Study

To determine the mechanism of the sorption process, an equilibrium study is very important. Sorption isotherms describe the relationship between the sorbate and the sorbent in the equilibrium state, the nature of the sorption process, the state of the sorbent surface, and give the sorption capacity of the sorbent [32,33]. Therefore, the experimental results obtained at the sorption equilibrium state were fitted using the Langmuir and Freundlich isotherm models, which are most commonly used to describe the mechanism of the sorption process. The results obtained by nonlinear regression analysis are shown in Figure S5, and the parameters of the isothermal models are shown in Table 4. The results obtained using the Langmuir model show significantly better agreement with the experimental results compared to the results of the Freundlich model. The determination coefficient for the Langmuir model is 0.95, while for the Freundlich model is 0.77. Also, the *MRD* values for these two models show that the agreement of the Langmuir model with the experimental results is significantly better. Therefore, it can be concluded that this is a monolayer sorption process without interaction between sorbed molecules, and it is assumed that sorption takes place at specific energetically homogeneous sites of the sorbent. The maximum sorption capacity obtained by the Langmuir isotherm model was 1051.11 mg g^−1^.

The dimensionless separation factor *R*_L_ decreases from 0.0108 to 0.0016 with the increase in initial dye concentration. The *R*_L_ value determines the spontaneity of the sorption process and since its value in the entire range of initial concentration of the RB19 dye is far less than 1, it can be concluded that the sorption process of the RB19 dye is a spontaneous process. Also, the sorption process is more favored at higher concentrations of RB19 dye. This can be attributed to the increase in the driving force of mass transfer between the liquid phase and the sorbent surface with the increase in the initial concentration of the RB19 dye.

#### 2.3.3. Thermodynamic Study

The thermodynamic parameters were calculated by using a linear regression analysis of the experimental results obtained at three different temperatures. The Δ*H*^0^ value was calculated from the slope, and the Δ*S*^0^ value from the intercept of the plot ln*K*_D_ versus *T*^−1^. The results are shown in Figure S6, and the values of thermodynamic parameters are shown in Table 4. A negative Δ*H*^0^ value indicates that the sorption has an exothermic nature, and Δ*H*^0^ value also gives information on the type of adsorption, which can be either physical or chemical. The chemisorption type is for Δ*H*^0^ values in the range of −80 kJ mol^−1^ to −400 kJ mol^−1^ [34]. Therefore, the obtained Δ*H*^0^ value indicates that the adsorption of RB19 on the BBN sorbent occurs by chemisorption. The positive value of Δ*S*^0^ indicates an increase in randomness at the solid/solution interface during the sorption of RB19 [35].

The positive values of Δ*G*^0^ at 5 °C indicate that the sorption process is non-spontaneous, while the negative values of Δ*G*^0^ at 15 °C and 25 °C indicate that the sorption process is spontaneous at those temperatures. The increase in Δ*G*^0^ with increasing temperature confirms that the sorption process is more favored at higher temperatures. The increase in Δ*G*^0^ with increasing temperature confirms that the sorption process is more favored at higher temperatures, and also that the dye binding occurs via chemisorption [36].

### 2.4. Reusability and Stability of Sorbent

**Figure 9:**
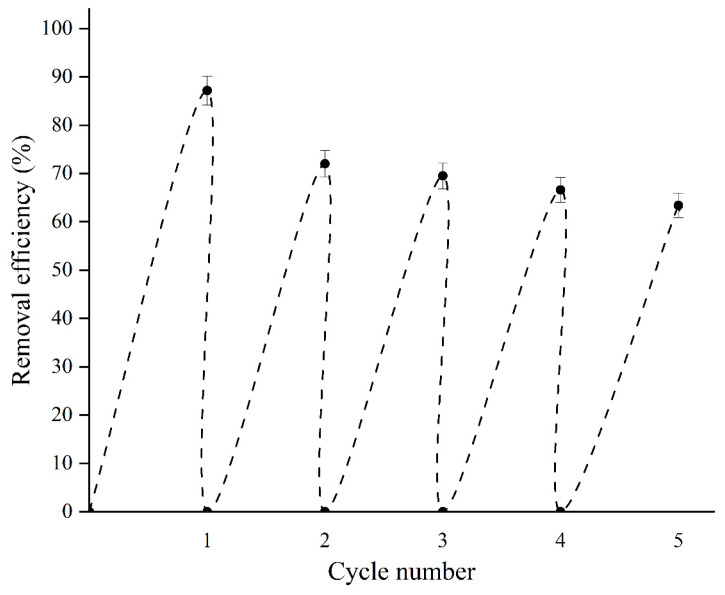
Removal efficiency of RB19 by BBN 200 mA cm^−2^ in five cycles: sorbent dose, 500.0 mg dm^−3^; initial RB19 concentration, 600.0 mg dm^−3^; stirring speed, 200 rpm; and temperature, 25.0 ± 0.5 °C.

For the successful practical application of sorbents, the reuse performance is of crucial importance. The reuse of material BBN 200 mA cm^−2^ has been investigated through consecutive sorption-desorption processes. As shown in Figure 5, the sorption performance decreases with the increase in pH value. Therefore, desorption was performed in an alkaline medium in a solution of NaOH. In an alkaline environment, negative OH^−^ ions most likely replace dye molecules bounded by the –SO_3_^−^ group on the sorbent surface, resulting in dye desorption. The sorption-desorption process was performed in five cycles. The parameters of the sorption process were the initial RB19 concentration of 600.0 mg dm^−3^, the sorbent dose of 500.0 mg dm^−3^ and the solution pH 2.0. Afterward, the sorption process material was removed from the solution by centrifugation at a stirring speed of 6000 rpm. To assess the stability of BBN 200 mA cm^−2^ sorbent the leached amount of bismuth in the supernatant was analyzed by using the ICP-OAS method. Then, desorption was performed by adding the used sorbent to a 0.1 mol dm^−3^ NaOH solution. The results are shown in Figure 9.

The results showed that after the first cycle, the dye removal efficiency decreases by 15%. After that, with each subsequent cycle, the removal efficiency slightly decreases by about 3–4%. The greater drop in removal efficiency after the first cycle can be explained by the permanent binding of the dye by chemisorption to the surface of the material. The slight drop in removal efficiency after the first cycle indicates that the material can be used for several cycles with high efficiency. Also, the material’s ability to desorb dye allows for the reuse of dye in the textile dyeing process.

Secondary contamination of water with bismuth from the BBN 200 mA cm^−2^ sorbent was examined after each cycle. The concentration of bismuth in the supernatant of all five cycles was under the limit of detection. This means that the sorbent is stable under sorption treatment conditions, and there is no contamination of water by bismuth.

### 2.5. The Mechanism of RB19 Sorption

Based on all the results of this manuscript, especially the characterization results, the influence of pH value, kinetics, isotherm, and thermodynamic studies, the possible sorption mechanism can be presented as follows. According to the XRD analysis, the chemical structure of the material is Bi_6_O_5_(OH)_3_(NO_3_)_5_⋅2H_2_O. Based on this structure of the material, it can be concluded that at low pH values, the surface of the material is positively charged, mostly like polycations [Bi_6_O_5_(OH)_3_]_5_^+^. In addition, in the acidic medium, the sulfonic group of the anionic dye RB19 is completely dissociated and, therefore, the most likely sorption mechanism is the chemical interaction between polycation [Bi_6_O_5_(OH)_3_]_5_^+^ and anionic functional group of dye –SO_3_^−^. Bands in the FTIR spectra characteristic of NO_3_^−^ and Bi–O vibrations confirm the structure of the material and the possible sorption mechanism. The results of the study of the influence of solution pH on the sorption capacity are consistent with the proposed sorption mechanism. The highest removal was achieved at pH 2.0 because under these conditions, the highest degree of protonation of the material and dissociation of the sulfonic groups of the RB19 dye is achieved. Therefore, it can be concluded that the sorption mechanism most likely involves electrostatic interaction, coordination bonds and chemical bonds formation. Kinetics, isotherms, and thermodynamic studies also show that the interaction between the sorbent and the RB19 dye is chemical in nature.

### 2.6. Comparative Analysis of Sorption Performance of Various Materials

In addition to the basic bismuth nitrates used in this study, various materials have been used to remove the RB19 dye by sorption, such as various biosorbents, activated carbons, biochar, and various inorganic sorbents. The results of the comparison of the maximum sorption capacity and the pH of the solution at which the maximum sorption capacity was achieved are shown in Table 5. Only ZnMnFe_2_O_4_ has a higher sorption capacity than BBN 200 mA cm^−2^, while the sorption capacity of other materials is significantly lower. Such sorption performances, along with the very short time it takes to reach the sorption equilibrium, make it a promising material for potential practical applications in water treatment.

## 3. Materials and Methods

### 3.1. Materials

All the chemicals used were of analytical reagent grade and used without further purification. The textile dye RB19 (chemical formula: C_22_H_16_N_2_Na_2_O_11_S_3_, type: anthraquinone dye, CAS number: 2580-78-1, color index number: 61200, molecular weight: 626.53 g mol^−1^, maximum absorbance: 592 nm) was supplied from Sigma–Aldrich (St. Louis, MO, USA). Nitric acid, sodium hydroxide, and ethanol were also purchased from Sigma–Aldrich. The bismuth (III) nitrate pentahydrate used for the synthesis of BBN was obtained from Acros Organics (Geel, Belgium). All the chemicals were of analytical grade and used as received without further purification. Deionized water was used for the preparation of all the solutions.

### 3.2. Procedure of Material Synthesis

Material synthesis was conducted by using an electrochemical procedure, i.e., cathodic electrodeposition from an acidic solution of bismuth nitrate and further thermal treatment of the obtained deposit. The solution from which the electrodeposition was carried out was obtained by dissolving bismuth nitrate in a nitric acid concentration of 1.0 mol dm^−3^. The bismuth nitrate concentration in the solution was 0.1 mol dm^−3^.

The electrodeposition was carried out in a two-electrode cell where a titanium sheet was used as the cathode (working electrode) and a stainless-steel sheet was used as the anode (counter electrode). The dimensions of both electrodes were 10 × 20 mm, and the distance between them was 15 mm. Before use, all electrodes were prepared by polishing with abrasive paper, degreasing with detergent, and then cleaning with ethanol and deionized water in an ultrasonic bath. An Amel 510 DC potentiostat–galvanostat (Material Mates, Rho (Milano), Italy) controlled by the VoltaScope 3.7 software package was used for the electrodeposition experiments. Electrodeposition was conducted at a constant current density. Then, the deposit obtained on the titanium electrode was thermally treated for 90 min. After the material cooled in air, it was removed from the titanium electrode, powdered, and used as such in all experiments.

### 3.3. Characterizations of Materials

For the SEM-EDX analysis, the samples were attached to aluminum stubs using agar carbon tabs. A JEOL5310LV (JEOL, Tokyo, Japan) was used for imaging the samples in low vacuum mode with an Oxford Instruments X-Max 50 detector for semiquantitative EDX analysis. The samples were imaged uncoated. Nominal magnifications from ×2000 to ×70,000 were used when imaging the samples. The three random particles were averaged for EDX analysis. The crystal structure was analyzed by XRD using filtered Cu Kα radiation (Ultima IV, Rigaku, Tokyo, Japan). The experiments were performed in the scan range 2*θ* = 5–90° under 40 kV and 40 mA with a scan speed of 5 degrees per min and steps of 0.02 degrees. Before the measurements were taken, angular correction was performed with a high-quality Si standard. Lattice parameters were refined from the data using the least square procedure. The standard deviation was about 1%. TG analysis was performed using a TGA K5000 (TA Instruments, New Castle, DE, USA). FTIR spectra were recorded in the range of 400–4000 cm^−1^ on Bomem MB-100 spectrometer (Hartmann & Braun, Québec, QC, Canada). The specific surface area was measured by nitrogen adsorption using a Gemini 5 Surface Area Analyzer (Micromeritics, Norcross, GA, USA). The samples were degassed under flowing nitrogen at 40 °C for 20 h before the measurements were taken. The specific surface area was determined using the Brunauer–Emmett–Teller (BET) method [46]. The Barret–Joyner–Halenda (BJH) method was used for pore volume, area, and diameter analysis [47]. The isoelectric point (pI) of the sorbent was determined by the salt addition method [48].

### 3.4. Batch Sorption Experiments

The sorption capacity of the obtained materials was determined by removing RB19 from the aqueous solution. Batch sorption experiments were carried out in flasks at ambient temperature using a magnetic stirrer at 200 rpm. The initial solution pH was adjusted by 0.1 mol dm^−3^ HNO_3_ and 0.1 mol dm^−3^ NaOH. Then, a sorbent was added to the solution at a dose of 500 mg dm^−3^. At certain time intervals, samples were taken and then filtered through 0.45 µm membrane filters (Agilent Technologies, Waldbronn, Germany) made of regenerated cellulose to remove sorbent particles. The residual concentration of RB19 in the samples was determined using a UV-1800 UV–Vis spectrophotometer (Shimadzu Corporation, Kyoto, Japan). The amount of sorbed dye (*q*_t_, mg g^−1^) and the removal efficiency (*RE*, %) were calculated by the following equations:

(1) $$q_{t}=\frac{c_{0}-c_{t}}{m}\cdot V$$

(2) $$RE(\%)=\frac{c_{0}-c_{t}}{c_{0}}\cdot 100$$

where *c*_0_ and *c*_t_ are the initial and final concentrations of RB19 (mg dm^−3^), respectively; *V* is the solution volume (dm^3^); and *m* is the mass of the sorbent (g).

The concentration of bismuth in the supernatant after the sorption treatment was determined using ICP-OES spectrometer iCAP 6500 Duo (Thermo Fisher Scientific, Waltham, MA, USA).

### 3.5. Kinetic, Isotherm and Thermodynamic Analysis

The kinetics of the sorption process were investigated using pseudo-first and pseudo-second order models. The nonlinear forms of these models can be represented by the following equations [49,50]:

(3) $$q_{t}={\;q}_{e}(1-e^{{-k}_{1}t})$$

(4) $$q_{t}=\frac{k_{2}q_{e}^{2}t}{1+k_{2}q_{e}t}$$

where *q*_t_ and *q*_e_ are sorption capacity at a certain time *t* and at the equilibrium state (mg g^−1^), respectively; *k*_1_ (min^−1^) and *k*_2_ (g mg^−1^ min^−1^) are sorption rate constants of pseudo-first and pseudo-second order model, respectively.

For the investigation of the sorption equilibrium, Langmuir and Freundlich sorption isotherms were utilized. The nonlinear forms of these models can be represented by the following equations [51,52]:

(5) $$q_{e}=\frac{q_{m}K_{L}c_{e}}{1+K_{L}c_{e}}$$

(6) $$q_{e}=K_{F}c_{e}^{\frac{1}{n}}$$

where *q*_m_ is maximum sorption capacity (mg g^−1^), *c*_e_ is equilibrium concentration of adsorbate (mg dm^−3^); *K*_L_ is Langmuir constant related to the sorption energy (dm^3^ mg^−1^); *K*_F_ is Freundlich equilibrium constant (mg g^−1^) (dm^3^ mg^−1^)^1/n^; *n* is Freundlich exponent related to the sorption intensity.

Separation factor *R*_L_, dimensionless constant, from the Langmuir isotherm is calculated from the equation:

(7) $$R_{L}=\frac{1}{1+K_{L}c_{0}}$$

The thermodynamics of the sorption process were investigated at temperatures of 5 °C, 15 °C, and 25 °C using the following equations [35]:

(8) $$K_{D}=\frac{c_{a}}{c_{e}}$$

(9) $$lnK_{D}=-\frac{∆H{}^{\circ}}{RT}+\frac{∆S{}^{\circ}}{R}$$

(10) $$∆G{}^{\circ}=-RTlnK_{D}$$

(11) ∆G°=∆H°−T∆S°

where Δ*H*^0^, Δ*S*^0^, and Δ*G*^0^ are the enthalpy, entropy, and Gibbs free energy of charge, respectively; *K*_D_ is constant of linear sorption distribution; *c*_e_ (mg dm^−3^) is dye concentration in the solution, and *c*_a_ (mg dm^−3^) is sorbed dye concentration.

The parameters of the kinetic and isothermal models were calculated by using a nonlinear regression analysis using the OriginPro 2021 software (OriginLab Corporation, USA). The determination coefficient (*R*^2^) in some cases is not the best for determining the degree of agreement between the results obtained by a certain model and the experimental results. Therefore, the mean relative deviation (*MRD*) was calculated for each nonlinear regression analysis according to the equation, as follows:

(12) $$MRD=\frac{\sum_{i=1}^{n}\left|q_{ti,\;\exp}-q_{ti,\;\mathrm{cal}}\right|}{\bar{q_{t,\;\exp}}n}\;\cdot \;100\%$$

where *q_ti_*_, exp_ is experimentally obtained sorption capacity (mg g^−1^) at experimental measurement *i* at a certain time *t* (min), *q_ti_*_, cal_ is sorption capacity (mg g^−1^) at experimental measurement *i* at a certain time *t* (min) calculated using a certain kinetic or isotherm model, $\bar{q_{t,\;\exp}}$ is mean value of sorption capacity of all experimental measurements (mg g^−1^), and *n* is number of experimental measurements.

## 4. Conclusions

In this study, for the first time, the influence of electrochemical synthesis parameters for obtaining compounds based on BBNs was examined. Based on the obtained results, it can be concluded that the parameters of the electrochemical synthesis have a huge influence on the morphology, crystal structure, and chemical structure of the synthesized materials. SEM analysis showed that with an increase in the current density during the process of electrodeposition, materials with lower crystallinity were obtained. XRD analysis revealed that the weight percentages of bismuth and bismuth oxide decreased, while the percentage of BBN increased. With an increase in the thermal treatment temperature to 500 °C, the largest percentage of Bi_6_O_5_(OH)_3_(NO_3_)_5_⋅2H_2_O oxidizes to Bi_2_O_3_, and the rest of the BBN changes its form to Bi_5_O_7_NO_3_. The structure of the obtained material was confirmed by FTIR and TG analysis. Additionally, the synthesis parameters strongly affect the sorption efficiency of BBN for the removal of RB19. The best sorption performance has the material obtained at 200 mA cm^−2^ at 200 °C. The cathode material has no influence on the morphology, crystal structure, chemical structure, or sorption properties of the obtained materials. Sorption of RB19 onto BBN sorbent following pseudo-second order model. Langmuir’s model has good agreement with the experimental results and can be used to describe the equilibrium state of the sorption process. The maximum sorption capacity according to the Langmuir model was 1051.10 mg g^−1^. Based on the thermodynamic parameters, it can be concluded that the binding process of the dye RB19 onto BBN sorbent has a chemisorption nature.

## Figures and Tables

**Table 1:** XRD analysis of the obtained materials.

	BBN 50 mA cm^−2^	BBN 100 mA cm^−2^	BBN 200 mA cm^−2^	BBN 350 °C	BBN 500 °C
Bi_6_O_5_(OH)_3_(NO_3_)_5_⋅2H_2_O (PDF 00-054-0627)	41.48%	72.28%	98.34%	/	/
Bi_2_O_3_ (PDF 01-074-1374)	30.51%	18.42%	1.67%	/	/
Bismuth (PDF 01-085-1329)	20.01%	9.30%	/	/	/
Bi_2_O_3_ (PDF 01-070-8243)	/	/	/	/	92.26%
Bi_5_O_7_NO_3_ (PDF 00-051-0525)	/	/	/	/	7.74%

**Table 2:** EDX analysis of the obtained materials.

	BBN 50 mA cm^−2^	BBN 100 mA cm^−2^	BBN 200 mA cm^−2^	BBN 350 °C	BBN 500 °C
Bi	75.5%	60.5%	66.2%	47.8%	72.7%
O	19.9%	31.2%	26.3%	25.6%	17.2%
N	3.5%	6.7%	6.2%	15.9%	/

**Table 3:** Textural properties of the obtained materials.

	BBN 50 mA cm^−2^	BBN 100 mA cm^−2^	BBN 200 mA cm^−2^	BBN 350 °C	BBN 500 °C
Surface area (m^2^ g^−1^)	0.6907	0.9787	0.956	0.1984	2.2319
Surface area of pores (m^2^ g^−1^)	0.691	0.927	0.962	0.200	2.276
Volume of pores (cm^3^ g^−1^)	0.001221	0.001499	0.00164	0.000414	0.003377
Average pore diameter (nm)	7.0665	6.4688	6.4166	8.2859	5.9344

**Table 4:** Kinetic, isotherm, and thermodynamic parameters for sorption of RB19 onto BBN 200 mA cm^−2^.

Kinetic Parameters						
*q*_e, exp_ (mg g^−1^) 1047.24						
Pseudo-first order			Pseudo-second order			
*q*_e, cal_ (mg g^−1^)	1032.47		*q*_e, cal_ (mg g^−1^)		1080.39	
*k*_1_ (min^−1^)	6.97		*k*_2_ (g mg^−1^ min^−1^)		0.016	
*R* ^2^	0.9975		*R* ^2^		0.9986	
*MRD* (%)	1.85		*MRD* (%)		0.69	
**Isotherm Parameters**						
*q*_e, exp_ (mg g^−1^) 1047.24						
Langmuir			Freundlich			
*q*_m_ (mg g^−1^)	1051.10		*K*_F_ (mg g^−1^) (dm^3^ mg^−1^)^1/n^		526.53	
*K*_L_ (dm^3^ mg^−1^)	1.03		*n*		6.60	
*R* ^2^	0.9548		*R* ^2^		0.7251	
*MRD* (%)	3.23		*MRD* (%)		17.65	
**Thermodynamic Parameters**						
	ln*K*_D_ (mg dm^−3^)	Δ*G*^0^ (kJ mol^−1^)	Δ*S*^0^ (J mol^−1^)	Δ*H*^0^ (kJ mol^−1^)	*R* ^2^	*MRD* (%)
Temperature 278.15 K	−1.32073	6.35	391.91	−112.08	0.9995	0.89
Temperature 288.15 K	0.34402	−2.37				
Temperature 298.15 K	1.93076	−4.9				

**Table 5:** Comparison of sorption performance of different materials for RB19 removal.

Material	pH	Sorption Capacity (mg g^−1^)	Ref.
ZnMnFe_2_O_4_	2.0	1498.99	[37]
BBN 200 mA cm^−2^	2.0	1051.10	This work
Lignin-based polyporous carbon@polypyrrole	2.0	537.52	[38]
Citrus waste activated carbon	natural pH	370.37	[39]
*Ulva reticulata* biochar	2.0	204.5	[40]
MgO nanoparticles	8.0	166.7	[41]
Starch modified NiFe-layered double hydroxide	6.31	149.4	[42]
Nano-carbon material	natural pH	116.01	[43]
NiO nanoparticles	6.5	98.83	[31]
*Citrus sinensis* biosorbent	2.0	75.19	[44]
Nanomagnetite supported biochar from *Eichhornia crassipes*	6.0	44.78	[45]

## Data Availability

The original contributions presented in this study are included in the article/Supplementary Materials. Further inquiries can be directed to the corresponding author(s).

## Supplementary Materials

The following supporting information can be downloaded at: https://www.mdpi.com/article/10.3390/molecules30051020/s1, Figure S1: TG curve of material synthesized at 200 mA cm^−2^ and 200 °C; Figure S2: TG curve of material synthesized at 200 mA cm^−2^ and 350 °C; Figure S3: TG curve of material synthesized at 200 mA cm^−2^ and 500 °C; Figure S4: Kinetic of RB19 sorption onto BBN 200 mA cm^−2^: initial RB19 concentration, 600.0 mg dm^−3^; sorbent dose, 500.0 mg dm^−3^; pH, 2.0; stirring speed, 200 rpm; temperature, 25.0 ± 0.5 °C; Figure S5: Isotherms of RB19 dye sorption onto BBN 200 mA cm^−2^: initial RB19 concentration, 600.0 mg dm^−3^; sorbent dose, 500.0 mg dm^−3^; pH, 2.0; stirring speed, 200 rpm; temperature, 25.0 ± 0.5 °C; Figure S6: Thermodynamic of RB19 dye sorption onto BBN 200 mA cm^−2^: initial RB19 concentration, 600.0 mg dm^−3^; sorbent dose, 500.0 mg dm^−3^; pH, 2.0; stirring speed, 200 rpm; temperature, 25.0 ± 0.5 °C.
