# Supplementary material for: Effect of Electrochemical Synthesis Parameters on the Morphology, Crystal and Chemical Structure, and Sorption Efficiency of Basic Bismuth Nitrates
Source: Molecules. 2025 Feb 23;30(5):1020. doi: 10.3390/molecules30051020 (PMC11901594; doi:10.3390/molecules30051020)
Supplement: Supplementary file 1 [file molecules-30-01020-s001.zip › molecules-3417992-supplementary.pdf]

Supplementary Materials

Effect of Electrochemical Synthesis Parameters on the Morphology, Crystal and Chemical Structure, and Sorption Efficiency of Basic Bismuth Nitrates

Slobodan M. Najdanović *, Miloš M. Kostić *, Milica M. Petrović, Nena D. Velinov, Miljana D. Radović Vučić, Jelena Z. Mitrović and Aleksandar Lj. Bojić

Department of Chemistry, Faculty of Sciences and Mathematics, University of Niš, Višegradska 33, 18000 Niš, Serbia; milica.petrovic3@pmf.edu.rs (M.M.P.); nena.velinov@pmf.edu.rs (N.D.V.); miljana.radovic@pmf.edu.rs (M.D.R.V.); jelena.mitrovic1@pmf.edu.rs (J.Z.M.); aleksandar.bojic@pmf.edu.rs (A.Lj.B.)

* Correspondence: najda89@gmail.com or slobodan.najdanovic@pmf.edu.rs (S.M.N.); mk484475@gmail.com or milos.kostic@pmf.edu.rs (M.M.K.)

Contents

Supplementary Figure S1: TG curve of material synthesized at 200 mA cm^{-2} and 200 °C.

Supplementary Figure S2: TG curve of material synthesized at 200 mA cm^{-2} and 350 °C.

Supplementary Figure S3: TG curve of material synthesized at 200 mA cm^{-2} and 500 °C.

Supplementary Figure S4: Kinetic of RB19 sorption onto BBN 200 mA cm^{-2}: initial RB19 concentration, 600.0 mg dm^{-3}; sorbent dose, 500.0 mg dm^{-3}; pH, 2.0; stirring speed, 200 rpm; temperature, 25.0 ± 0.5 °C.

Supplementary Figure S5: Isotherms of RB19 dye sorption onto BBN 200 mA cm^{-2}: initial RB19 concentration, 600.0 mg dm^{-3}; sorbent dose, 500.0 mg dm^{-3}; pH, 2.0; stirring speed, 200 rpm; temperature, 25.0 ± 0.5 °C.

Supplementary Figure S6: Thermodynamic of RB19 dye sorption onto BBN 200 mA cm^{-2}: initial RB19 concentration, 600.0 mg dm^{-3}; sorbent dose, 500.0 mg dm^{-3}; pH, 2.0; stirring speed, 200 rpm; temperature, 25.0 ± 0.5 °C.

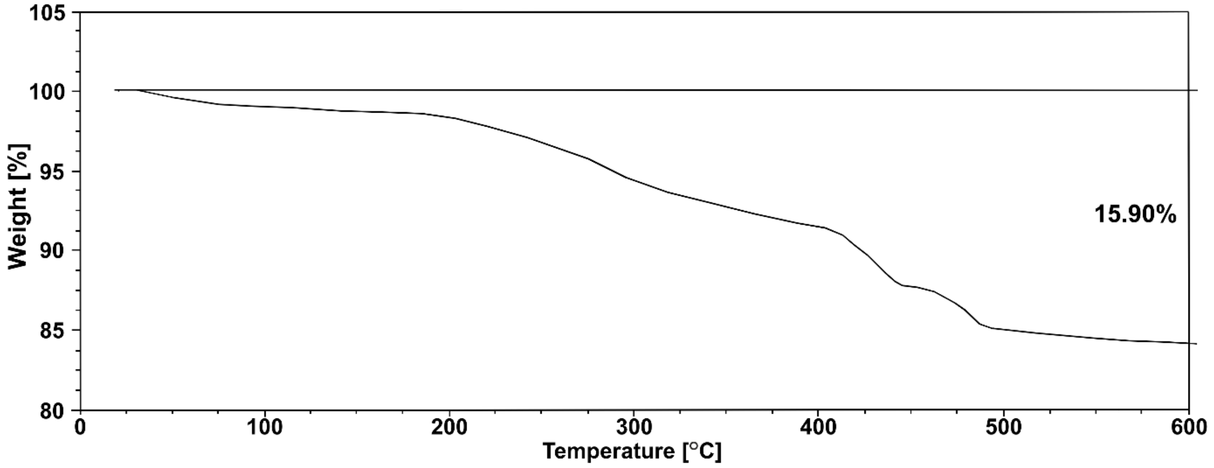

Supplementary Figure S1.

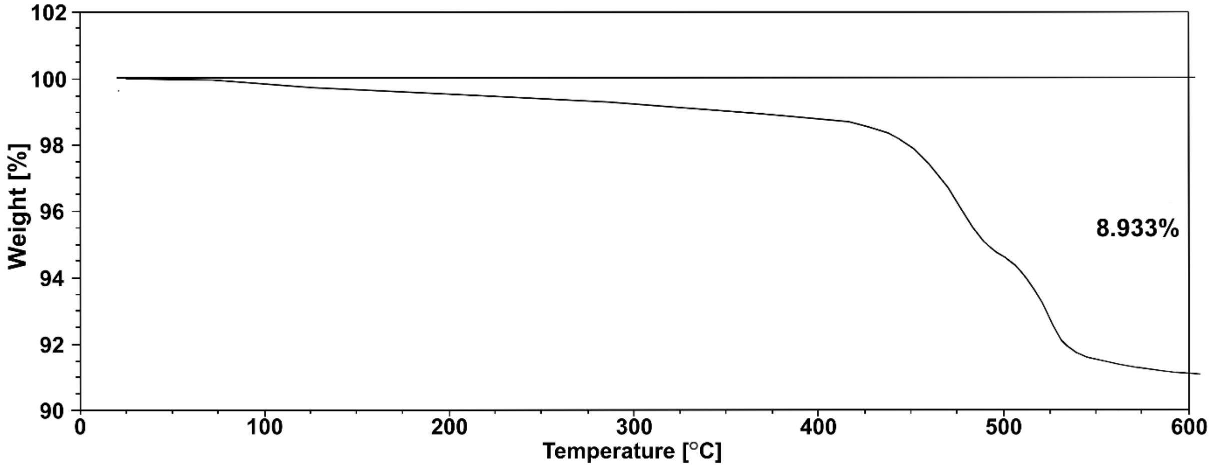

Supplementary Figure S2.

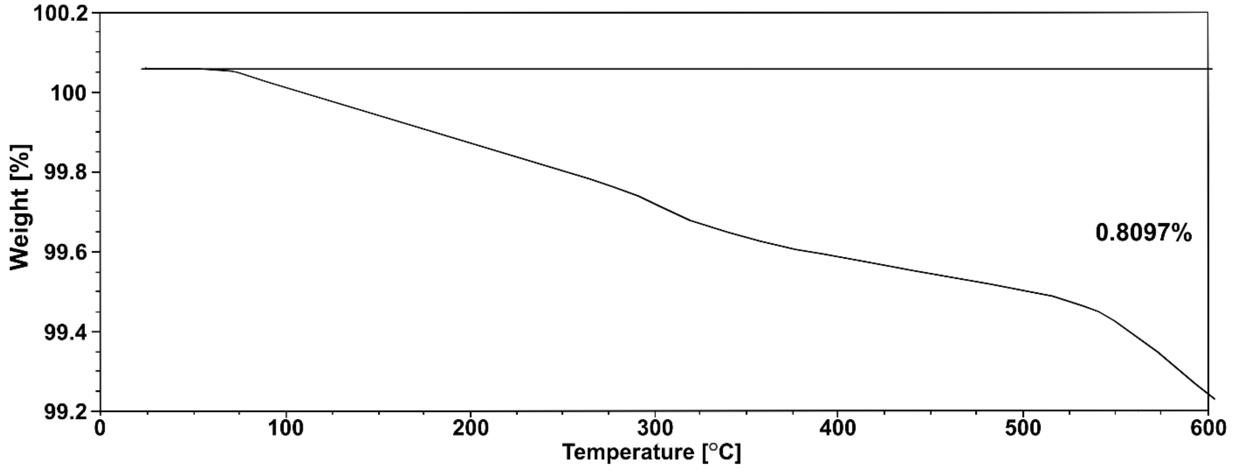

Supplementary Figure S3.

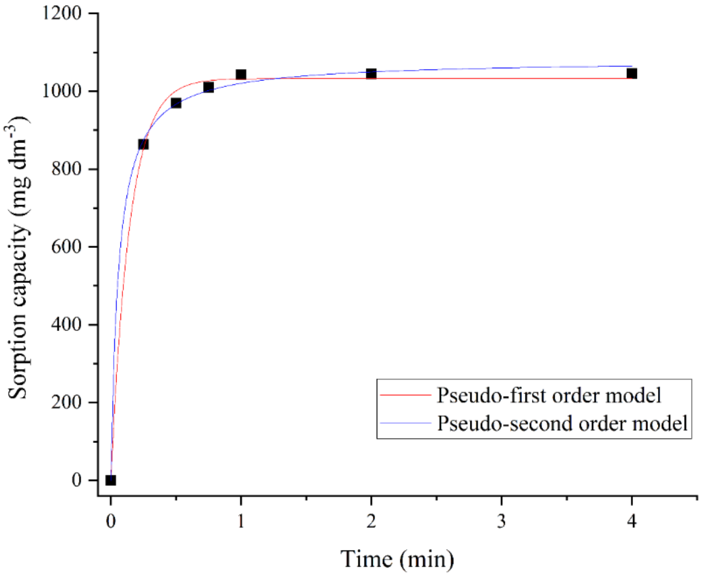

Supplementary Figure S4.

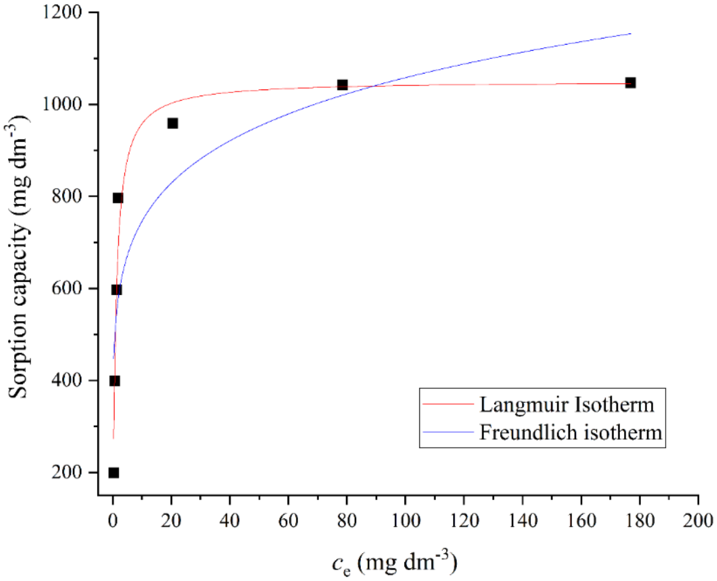

Supplementary Figure S5.

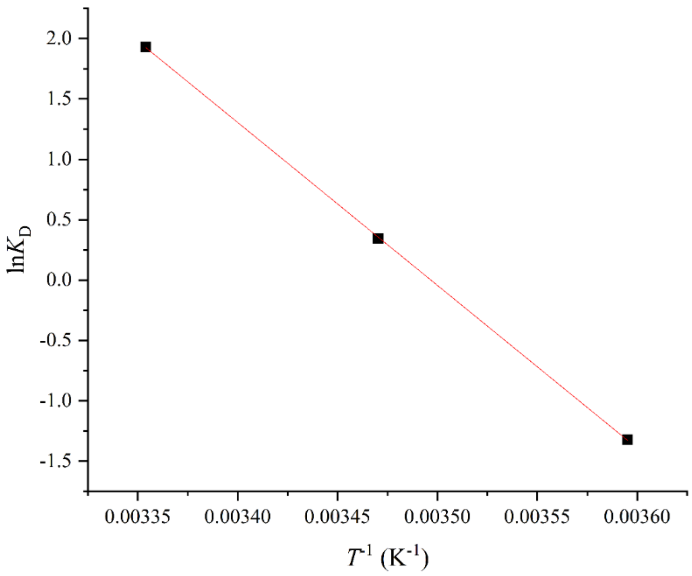

Supplementary Figure S6.
